# Supplementary material for: An adaptable analysis workflow for characterization of platelet spreading and morphology
Source: Platelets. 2020 Apr 23;32(1):54–8. doi: 10.1080/09537104.2020.1748588 (PMC8802896; doi:10.1080/09537104.2020.1748588)
Supplement: Supplemental Material [file IPLT_A_1748588_SM0817.pdf]

Supplementary methods

Platelet preparation and treatment

Platelets were isolated from human blood samples, which were donated by healthy volunteers. Human venous blood was drawn by venipuncture into sodium citrate and acid-citrate-dextrose solution. Whole blood was centrifuged at 200 × g for 20 minutes to obtain platelet rich plasma. 0.1 µg/ml prostacyclin was added to the platelet rich plasma and platelets were collected after centrifugation at 1000 × g for 10 minutes. The platelet pellet was re-suspended in acid-citrate-dextrose and modified Tyrode's buffer containing 129 mM NaCl, 0.34 mM Na2HPO4, 2.9 mM KCl, 12 mM NaHCO3, 20 mM HEPES, 5 mM glucose, 1 mM MgCl2, pH 7.3 and 0.1 µg/ml prostacyclin was added to the washed platelets and centrifuged at 1000 × g for 10 minutes to be washed. The platelet pellet was re-suspended in modified Tyrode's buffer to a concentration of 2×10^8 platelets/ml and left to rest for at least 30 minutes before further dilution to 2×10^7 platelets/ml prior to being used in spreading experiments.

Coverslips were coated with 100 µg/ml fibrinogen or 10 µg/ml collagen and left overnight at 4 ºC. The unbound fibrinogen and collagen were removed and the coverslips were blocked using 5 mg/ml bovine serum albumin (BSA) in PBS for 1 hour and washed with PBS prior to use. All spreading experiments were performed in the presence of 2 U/ml apyrase and 10 µM indomethacin. 2×10^7 platelets/ml washed platelets were either incubated for 10 minutes at 37 ºC in the presence of 10 µM dasatinib or DMSO, prior to 45 minutes of spreading on pre-coated coverslips at 37 ºC.

Imaging

Platelets were fixed after spreading using 10% neutral buffered formalin solution for 10 minutes at room temperature. 0.1% Triton X-100 was added for 5 minutes at room temperature prior to platelets being washed with PBS and incubated with Alexa488-phalloidin for 1 hour at room temperature to stain the filamentous (F)-actin fibres present within the platelets for the

observation of platelet morphology. The actin stained platelets were mounted onto glass slides using hydromount. Images were acquired using an Axio Observer 7 inverted epifluorescence microscope (Carl Zeiss Microscopy) with Definite Focus 2 autofocus, 63x 1.4 NA oil immersion objective lens, Colibri 7 LED illumination source, Hamamatsu Orca Flash 4 V2 sCMOS camera, Filter set 38 for Alexa488 and DIC optics. LED power and exposure time were chosen as appropriate for each set of samples but kept the same within each experiment. Using Zen 2.3 Pro software. Three image stacks (step size 0.3 µm) were taken per coverslip.

Segmentation performance evaluation

To evaluate the performance of the proposed segmentation workflow, 12 cropped images (one for each condition and replicate) were manually annotated by drawing around the boundary of each cell (Supplementary Figure 1). This was done using the Labelbox Image Segmentation web application.

Segmentation performance was quantified using the methodology developed for the Cell Tracking Challenge[1]. Each reference manually outlined cell, *R*, was compared to each segmented cell, *S*. The reference cell was said to match a segmented cell if the union of *R* and *S* was greater than 50% the area of *R*. For each reference cell the Jaccard Index was calculated as the intersection of the reference and corresponding matched cell divided by their union;

$$J(R) = \frac{|R \cap S|}{|R \cup S|}$$

If no match was found then $J(R) = 0$. The Jaccard index was then averaged for all reference cells in the image (Supplementary Figure 2).

For comparison a simple fully automated workflow was implemented and compared to the manual segmentations with the same evaluation methodology. First a Gaussian blur (sigma set to 2 pixels) was applied which was followed by an automated Otsu threshold[2]. Holes in the resulting binary image were filled. A watershed transform was then used to try and separate touching platelets. Performance was evaluated with, and without, the watershed transform

(Supplementary Figures 1 and 2). In both cases, and in alignment with the proposed workflow, cells smaller than 4 μm^2 were removed from the segmentation result. These simple workflows were automated using ImageJ macros[3].

Morphological features for object classification

Object classifiers were trained using simple morphological features which were calculated using the Image Segment Features KNIME node. Specifically area, circularity, perimeter, convexity, extend and diameter. Circularity is defined as $4\pi \times \frac{Area}{Perimeter^2}$ such that a value of 1 indicates a perfect circle.

Supplementary references

1. Maška M, Ulman V, Svoboda D, Matula P, Matula P, Ederra C, Urbiola A, España T, Venkatesan S, Balak DM, others. A benchmark for comparison of cell tracking algorithms. *Bioinformatics*. 2014;30:1609–1617.

2. Otsu N. A threshold selection method from gray-level histograms. *Automatica*. 1975;11:23–27.

3. Schindelin J, Arganda-Carreras I, Frise E, Kaynig V, Longair M, Pietzsch T, Preibisch S, Rueden C, Saalfeld S, Schmid B, others. Fiji: an open-source platform for biological-image analysis. *Nature methods*. 2012;9:676–682.

Supplementary Figures

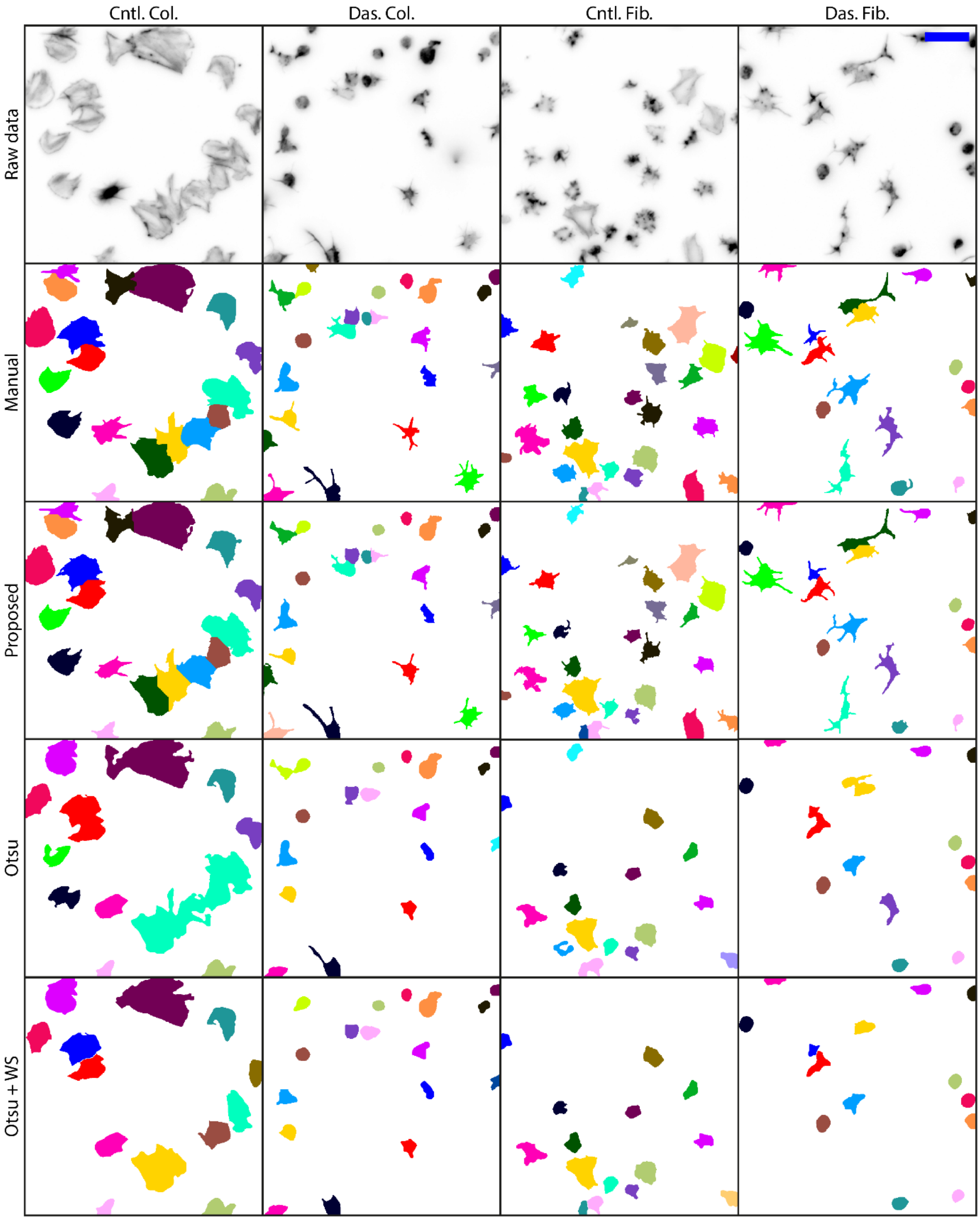

Supplementary Figure 1. Representative images and segmentation results from platelets seeded on either collagen (Col.) or fibrinogen (Fib.) and treated with either dasatinib (Das.) or a DMSO control (Cntl.). Top row (raw data) shows a maximal projection of the raw data

(inverted grey-scale look-up-table). The manual segmentations are the result of a researcher manually outlining each platelet. This provides a reference for the performance evaluation. The third row shows the results of the proposed semi-automated workflow. The bottom two rows show the results from a simple fully-automated non-machine learning based analysis based on Otsu thresholding either without (Otsu), or with (Otsu + WS), watershed post-processing for object separation. Scale bar 10 µm.

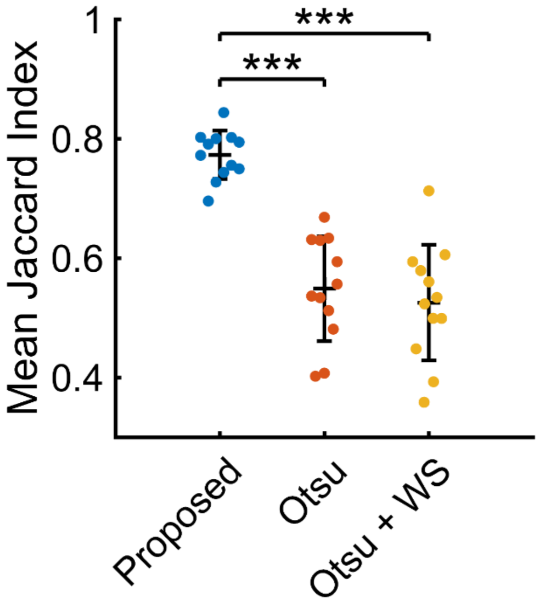

Supplementary Figure 2. Segmentation performance of the proposed method and simple fully-automated analysis worfklows based on Otsu thresholding either without (Otsu), or with (Otsu + WS), watershed post-processing for object separation. The Jaccard Index is calculated for each manually annotated reference cell and averaged for all cells in an image. The proposed method works accurately and consistently in contrast to the simple fully automated workflows. A single cropped image from each condition and replicate were evaluated (12 images and 220 cells total). Statistical analysis by one-way Anova and subsequent pair-wise comparison by two-sample t-test with Bonforonni correction. ***P<0.001, error bars are mean ± s.d.

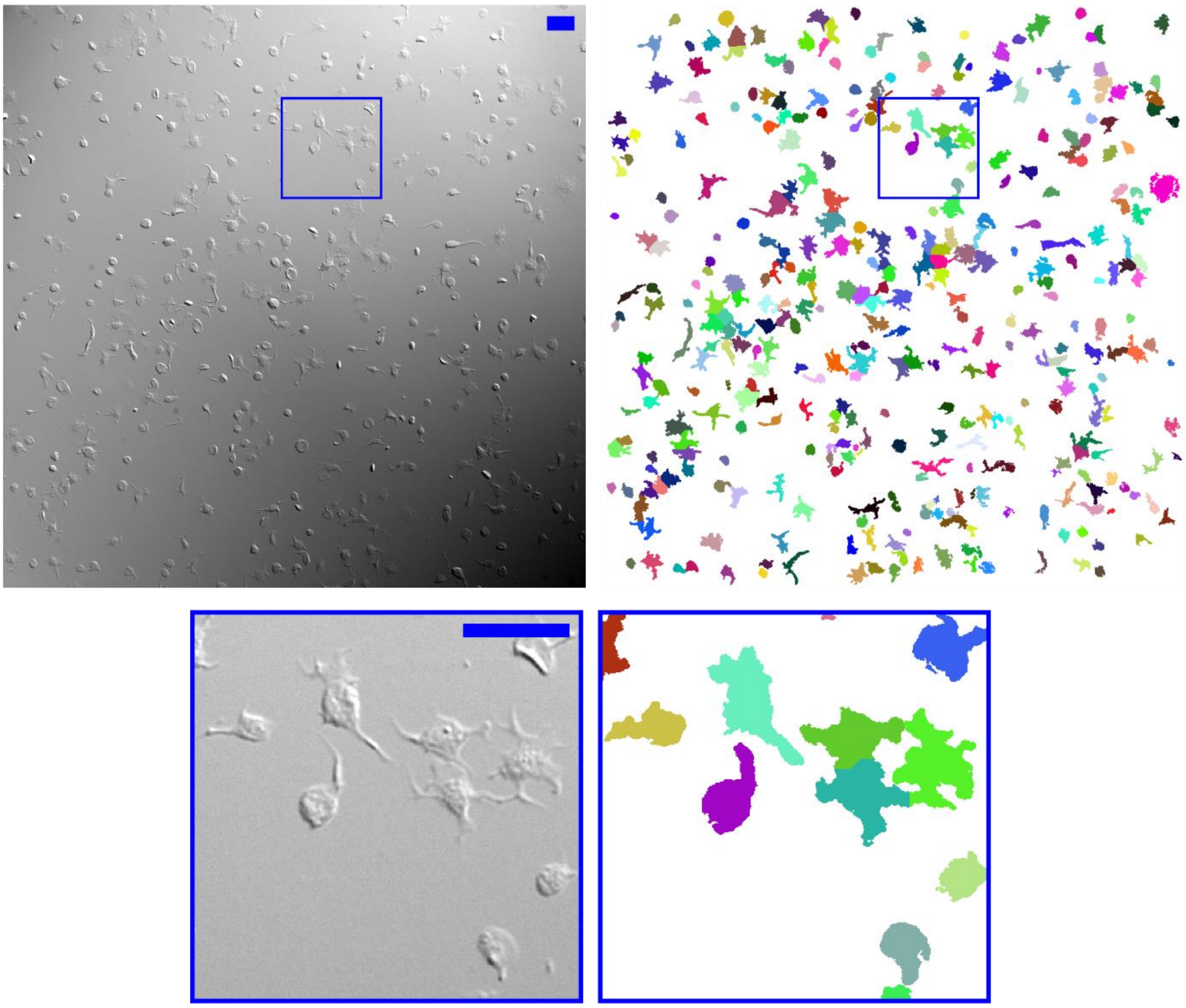

Supplementary Figure 3. Phase contrast images and segmentation results for a representative acquisition where platelets were spread on fibrinogen. Full field of view and cropped images are shown. Each cell segmentation is represented by a different colour. The workflow is able to produce reasonable segmentation without using a florescent stain. Scale bar 10 µm.
